# Causal relationship between *Butyricimonas* and allergic asthma: a two-sample Mendelian randomization study

**DOI:** 10.3389/fmicb.2023.1190765

**Published:** 2023-10-06

**Authors:** Wei Wan, Yi Qiu, Xinyi Huang, Yingxiang Ren, Andi Peng, Chunping Yang

**Affiliations:** Department of Otorhinolaryngology Head and Neck Surgery, The Second Affiliated Hospital of Nanchang University, Nanchang, China

**Keywords:** allergic asthma, allergic diseases, causal relationship, gut microbial genera, Mendelian randomization

## Abstract

**Background:**

Growing evidence has well documented the close association between the gut microbiome and allergic respiratory disease, which has been notably represented by allergic asthma. However, it is unclear whether this association is a causal link. Therefore, we investigated the potential causal associations between the gut microbiome and allergic asthma or other allergic diseases.

**Methods:**

In this study, we performed two-sample Mendelian randomization (MR) analyses by using the publicly available genome-wide association study (GWAS) summary data. Single-nucleotide polymorphisms (SNPs) that significantly correlated were selected as instrumental variables. The inverse variance weighted (IVW) method was used to examine the potential causal gut microbial genera for allergic asthma and other allergic diseases. The robustness of the primary findings of the MR analyses was ensured by using different sensitivity analyses.

**Results:**

Combining the findings from multiple analyses, the host genetic-driven increases in *Butyricimonas* at the genus level were positively correlated with the risk of allergic asthma. In addition, phylum *Bacteroidetes* and class *Bacteroidia* were also found to have negative associations with the risk of allergic asthma; genus *Slackia* was identified as having potential causal effects with allergic asthma. No clear evidence of pleiotropy and heterogeneity was observed in genus *Butyricimonas*. *Butyricimonas* was also found to have an association with allergic rhinitis, but not with other allergic diseases.

**Conclusion:**

Our findings indicate that there are new gut microbial genera that were causally associated with the risk of allergic asthma and other allergic diseases, and offer novel insights into the pathogenesis of allergic respiratory diseases.

## 1. Introduction

Allergic asthma is the most common phenotype of asthma. These patients are usually allergic to aeroallergens from the environment. After repeatedly inhale allergens, leading to acute bronchoconstriction, mediating the immune response produced by IgE and cytokines, promoting mast cells release mediators and recruitment of other cell types, which cause a late asthmatic response (Lotvall et al., 2011; Schatz and Rosenwasser, 2014; Akar-Ghibril et al., 2020). Allergic asthma is often accompanied by allergic rhinitis and conjunctivitis (Verschoor and von Gunten, 2019). Genetic, environmental and stress factors interact in susceptible individuals, resulting in changes in their immune system and enhanced airway responsiveness (Gaffin and Phipatanakul, 2009; Haczku and Panettieri, 2010; Thomsen, 2015). Presently, the treatments for allergic asthma mainly include environmental control measures, allergen immunotherapy, and anti-inflammatory inhaled corticosteroids (ICS). There are still some patients with allergic asthma may require sustained systemic corticosteroids to control their symptoms. These patients will be away from work or school for several days during the onset of allergic asthma and are at risk from the many potential serious side effects of long-term corticosteroid treatment. Therefore, it is imperative to explore the etiology of allergic asthma in order to promote the development of treatment strategies with low damage or even no side effects.

Recently, the causal relationship between the gut microbiome and allergic asthma risk has attracted widespread attention (Smith et al., 2013; Stiemsma and Turvey, 2017; Barcik et al., 2020). Many recent studies had showed that dysbiosis of gut microbiome may lead to the development of allergic disease, as gut microbiome has a significant impact on the maturation of immune cells and resistance to pathogens early (Azad et al., 2013). The gut microbiome seems to be a potential therapeutic target for preventing allergic asthma and other allergic diseases (Atarashi et al., 2011; Cukrowska et al., 2020; Wu et al., 2022). Several studies had shown that allergic asthma patients have dysbiosis of gut microbiome, with a significantly decreased in the relative abundance of some gut microbiome (Fujimura et al., 2016; Arrieta et al., 2018). Metabolites of gut microbiome can also have an impact on allergic asthma. Short-chain fatty acids (SCFAs) are metabolites produced by symbiotic bacteria in the gastrointestinal tract, maintained by fermentation of indigestible digestive products and intestinal amino acids, and they can be absorbed into the circulatory system (Le Poul et al., 2003; Trompette et al., 2014). In both ovalbumin (OVA)- and house dust mite (HDM)-induced models of allergic asthma, SCFAs have been documented to reduce inflammation (Trompette et al., 2014; Cait et al., 2018). However, the human gut microbiome can also produce pro-inflammatory metabolites, such as histamine (Pugin et al., 2017). The number of histamine-secreting bacteria in fecal samples of patients with allergic asthma is significantly higher, which correlates with the severity of the disease (Barcik et al., 2019). The guidelines issued by the World Allergy Organization (WAO) in 2015 indicated that use gut probiotics in not-exclusively breastfed infants places a relatively higher value on possible prevention of allergies (Cuello-Garcia et al., 2016), it can also reduce the risk of developing recurrent wheezing, which has been widely explored in many randomized controlled trials (RCT) (Ivakhnenko and Nyankovskyy, 2013; Moossavi et al., 2018). A meta-analysis involving 19 RCTs found that probiotics could significantly reduce wheeze incidence among infants with allergic asthma (Wei et al., 2020). Another meta-analysis involving 17 RCTs found that gut probiotics could significantly reduce the risk of eczema (Chen et al., 2022). Eczema is the earliest allergic reaction that occurs in childhood and is considered an early sign of a series of allergic diseases, such as allergic rhinitis and allergic asthma (Silverberg and Simpson, 2013; Chen et al., 2022). Although this study has not yet found that supplementation of gut microbiome in infants has a significant preventive effect on allergic asthma and allergic rhinitis, it demonstrates that the gut microbiome is important for forming the immune system and establishing the cell balance. Even so, these evidences are contradictory, they all prove that the gut microbiome is indeed involved in the immune regulation of allergic asthma, but the mechanism is still unclear. The causal association between gut microbiome and allergic asthma has not been fully confirmed.

Mendelian randomization (MR) uses instrumental variables (IVs) and is a common method that is used to examine whether there is a causal association between exposure and complex outcomes (Liu et al., 2022). The results of MR analysis mainly depend on the aggregated data of the genome-wide association studies (GWAS) database, thus greatly reducing the impact of confounding factors. Therefore, in this study we used the most up-to-date GWAS database to conduct an MR analysis to investigate the potential causal associations between gut microbial genera and the risk of allergic diseases including allergic asthma, allergic rhinitis, allergic purpura, allergic conjunctivitis, allergic contact dermatitis, and allergic urticaria (Bernstein et al., 2014; Gaudinski and Milner, 2017).

## 2. Materials and methods

### 2.1. Study design

**FIGURE 1:**
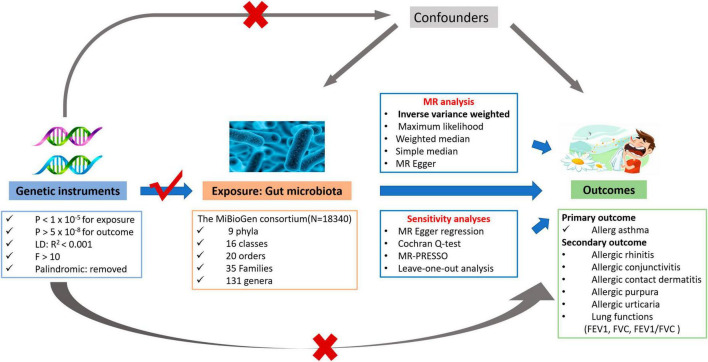
The overview flowchart of hypothesis and study design. This study was a two-sample Mendelian randomization analysis testing the causal effects between gut microbiome and allergic asthma. There are three assumptions required for a valid genetic instrument: the SNPs were significantly correlated with gut microbiome, they were independent of confounders, and the allergic asthma was only related through gut microbiome.

The overall flowchart for this study is illustrated in Figure 1. We conducted a two-sample MR study to investigate the causal relationships between host genetic-driven gut microbiome and allergic asthma. For significant results, we then explored whether associations exist among the gut microbiome, lung functions (FEV1, FVC, and FEV1/FVC ratio), and five allergic diseases, namely, allergic rhinitis, allergic purpura, allergic conjunctivitis, allergic contact dermatitis, and allergic urticaria.

### 2.2. Data sources and SNP selection

Summary genetic association estimates for the human gut microbiome were obtained from the largest, genome-wide meta-analysis, the MiBioGen consortium study (Kurilshikov et al., 2021), which consisted of 18,340 individuals from 24 population-based cohorts of European (*n* = 13266), Hispanic, Middle Eastern, Asian, and African ancestries. In addition, the GWAS study was adjusted for sex, age, genetic principal components, and cohort-specific potential microbiome batch effects.

Genome-wide association study summary statistics for allergic asthma and five other allergic diseases were drawn from the FinnGen consortium round 8 databases (Supplementary Table 1) after adjusting for sex, age, 10 principal components, and genotyping batch. FinnGen is a large public-private partnership aiming to collect and analyze genome and health data from 500,000 Finnish biobank participants. The diagnoses were based on ICD-10 and the following study IDs were used to obtain the outcome data: allergic asthma, allergic rhinitis, allergic purpura, allergic conjunctivitis, allergic contact dermatitis and allergic urticaria.

For lung function, we focused on forced expiratory volume in 1-second (FEV1), forced vital capacity (FVC), and FEV1/FVC ratio (Supplementary Table 1). The related summary statistics were extracted from UK Biobank, comprising a sample size of 344,267 participants of European ancestry, which were then adjusted for age, age*age, gender, height, and smoking status.

Based on the previously published studies and getting more acceptable instrumental variables (Sanna et al., 2019), the SNPs association threshold was set to be *p* < 1.0 × 10^–5^, but it was not associated with the outcome (*p* > 5.0 × 10^–8^). A total of five levels, namely, phylum, class, order, family, and genus, of bacterial taxa were analyzed in our study. Then, to retain the independent instrumental variables, SNPs were clumped and discarded at linkage disequilibrium *r*^2^ < 0.001 within a 10,000 kilobase pairs window, which was based on reference data of European ancestry from the 1000 Genomes Project (Genomes Project et al., 2015). Subsequently, weak instrumental variables were removed using the F statistic (*F* < 10). Finally, after harmonizing the exposure and the outcome data, the palindromic SNPs (A/T, G/C) were also excluded from the MR analysis.

### 2.3. Mendelian randomization analysis and sensitivity analyses

A two-sample MR analysis was conducted using the inverse-variance weighted (IVW) method as the primary analysis. IVW is classically used in combination with the Wald ratio estimates to obtain an unbiased estimate (Burgess et al., 2013). To provide robust evidence of results, four additional methods were employed to validate the findings, namely, the maximum likelihood method (Burgess et al., 2015), weighted median (Bowden et al., 2016), simple median (Burgess et al., 2016), and MR–Egger method (Bowden et al., 2015). The MR–Egger regression analysis allows free evaluation of the non-zero intercept value as an estimated value of average pleiotropic bias, and it gives a consistent estimate even if all of the instrumental variables are invalid. If some instrumental variables were valid, an unbiased estimate of causality could be provided by the weighted median method and maximum likelihood method.

To ensure the robustness of MR findings, The MR–Egger intercept test and Cochrane’s Q heterogeneity method were conducted to detect any potential horizontal pleiotropy (Higgins and Thompson, 2002). We also performed the Pleiotropy Residual Sum and Outlier (MR–PRESSO) test to find and correct the outlier instrumental variables reflecting pleiotropic biases (Verbanck et al., 2018). Additionally, we applied the leave-one-out method to rule out the possibility of the influence of a single SNP on the causality.

### 2.4. Statistical analysis

All of the analyses were conducted using the TwoSampleMR package and MR–PRESSO package in R software (version 4.1.2). The MR results are presented as odds ratios (ORs) with 95% confidence intervals (CIs). The significant threshold for each level was corrected based on the Bonferroni’s method (*p* < 0.05/n). A value of *p* < 0.05 but above the Bonferroni’s–corrected significance threshold was considered suggestive of evidence for a potential causal effect.

## 3. Results

Under the suggestive significance threshold of *p* < 1.0 × 10^–5^, a total of 1,958 independent SNPs from 175 bacterial taxa (excluding the unknown taxa) were identified after a series of quality control. The 175 bacterial taxa consisted of 11 classes, 26 families, 117 genera, 12 orders, and 9 phyla. To account for multiple testing, we implemented the Bonferroni’s-corrected method to examine for associations with allergic asthma risks using the IVW model, and the results are listed as follows: class *p* < 4.5 × 10^–3^ (0.05/11), family *p* < 1.9 × 10^–3^ (0.05/26), genus *p* < 4.2 × 10^–4^ (0.05/117), order *p* < 4.2 × 10^–3^ (0.05/12), and phylum *p* < 5.6 × 10^–3^ (0.05/9).

### 3.1. Causal associations of *Butyricimonas* with allergic asthma

Based on the IVW model, the host genetic-driven increases in *Butyricimonas* at the genus level were positively associated with a higher risk of allergic asthma (OR = 1.279, 95% CI = 1.12–1.46, *p* = 2.87 × 10^–4^, Table 1). The conclusion was confirmed by the results of the maximum likelihood method (OR = 1.283, 95% CI = 1.12–1.47, *p* = 3.24 × 10^–4^), weighted median method (OR = 1.31, 95% CI = 1.098–1.563, *p* = 2.74 × 10^–3^), and simple median method (OR = 1.27, 95% CI = 1.053–1.532, *p* = 1.24 × 10^–2^). In addition, four suggestive causal effects were identified, within the phylum *Bacteroidetes* (OR = 0.8, 95% CI = 0.673–0.951, *p* = 1.14 × 10^–2^), class *Bacteroidia* (OR = 0.819 95% CI = 0.695–0.965, *p* = 1.69 × 10^–2^), and genus *Slackia* (OR = 1.169, 95% CI = 1.021–1.34, *p* = 2.42 × 10^–2^). The details on instrumental variables are summarized in Supplementary Table 2.

**TABLE 1:** Mendelian randomization (MR) results of gut microbiome on allergic asthma.

Level	Exposure	Outcome	Method	Nsnp	OR (95% CI)	*P*	MR Egger regression		Cochran *Q*-test		MR-PRESSO
							**Egger intercept**	**P**	**Q**	**P**	**Global Test P**
Genus	Butyricimonas	Allergic asthma	Inverse variance weighted	15	1.279 (1.12–1.46)	2.87E-04	0.009	0.654	6.485	0.927	0.952
			Maximum likelihood	15	1.283 (1.12–1.47)	3.24E-04					
			Weighted median	15	1.31 (1.098–1.563)	2.74E-03					
			Simple median	15	1.27 (1.053–1.532)	1.24E-02					
			MR Egger	15	1.148 (0.71–1.855)	5.74E-01					
Phylum	Bacteroidetes	Allergic asthma	Inverse variance weighted	13	0.8 (0.673–0.951)	1.14E-02	−0.007	0.67	6.63	0.828	0.874
			Maximum likelihood	13	0.795 (0.666–0.95)	1.17E-02					
			Weighted median	13	0.884 (0.698–1.118)	3.03E-01					
			Simple median	13	0.852 (0.668–1.087)	1.98E-01					
			MR Egger	13	0.87 (0.575–1.317)	5.11E-01					
Class	Bacteroidia	Allergic asthma	Inverse variance weighted	15	0.819 (0.695–0.965)	1.69E-02	−0.005	0.73	10.131	0.683	0.773
			Maximum likelihood	15	0.818 (0.691–0.969)	1.98E-02					
			Weighted median	15	0.887 (0.705–1.116)	3.06E-01					
			Simple median	15	0.851 (0.671–1.08)	1.84E-01					
			MR Egger	15	0.876 (0.58–1.323)	5.30E-01					
Genus	Slackia	Allergic asthma	Inverse variance weighted	8	1.169 (1.021–1.34)	2.42E-02	−0.0007	0.988	6.698	0.35	0.471
			Maximum likelihood	8	1.177 (1.022–1.356)	2.36E-02					
			Weighted median	8	1.159 (0.958–1.402)	1.30E-01					
			Simple median	8	1.164 (0.973–1.393)	9.67E-02					
			MR Egger	8	1.177 (0.516–2.686)	6.99E-01					

**FIGURE 2:**
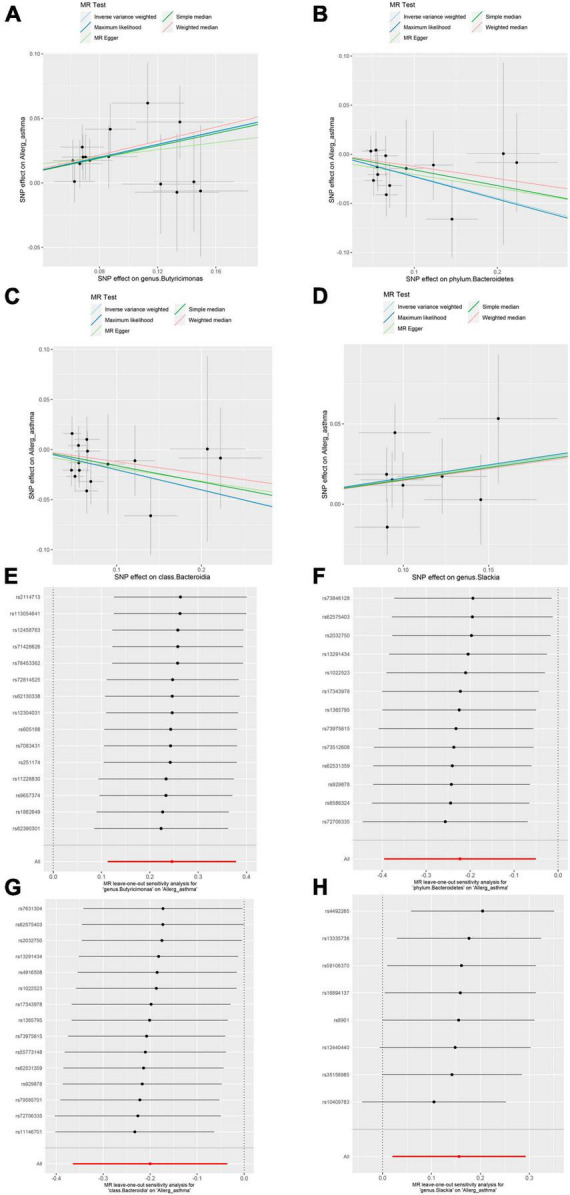
Mendelian randomization (MR) sensitivity analysis for gut microbiome on allergic asthma. Scatterplot of five MR tests between the genus *Butyricimonas* **(A)**, phylum *Bacteroidetes* **(B)**, class *Bacteroidia* **(C)** and genus *Slackia* **(D)** and allergic asthma. MR leave-one-out sensitivity analysis are included for the genus *Butyricimonas* **(E)**, phylum *Bacteroidetes* **(F)**, class *Bacteroidia* **(G)**, and genus *Slackia* **(H)** on allergic asthma.

In the sensitivity analysis, no clear evidence of pleiotropy (MR–Egger intercept = 0.009, MR–Egger *p* = 0.654, and MR–PRESSO Global Test *p* = 0.952) and heterogeneity (Cochran *Q* = 6.485, *p* = 0.927) was observed in genus *Butyricimonas* (Table 1). The results of leave-one-out sensitivity analysis revealed that no single SNP drives the causal associations between the genus *Butyricimonas* and allergic asthma (Figure 2). Furthermore, no evidence of heterogeneity or pleiotropy bias was noted among the phylum *Bacteroidetes*, class *Bacteroidia*, and genus *Slackia* as well (Figure 2). Thus, the two-sample MR estimates found that the genus *Butyricimonas* was positively related to allergic asthma risk, phylum *Bacteroidetes* and class *Bacteroidia* were negatively related to allergic asthma risk, and genus *Slackia* was identified as having potential causal effects with allergic asthma (Table 1).

### 3.2. No causal association of *Butyricimonas* with lung function

**FIGURE 3:**
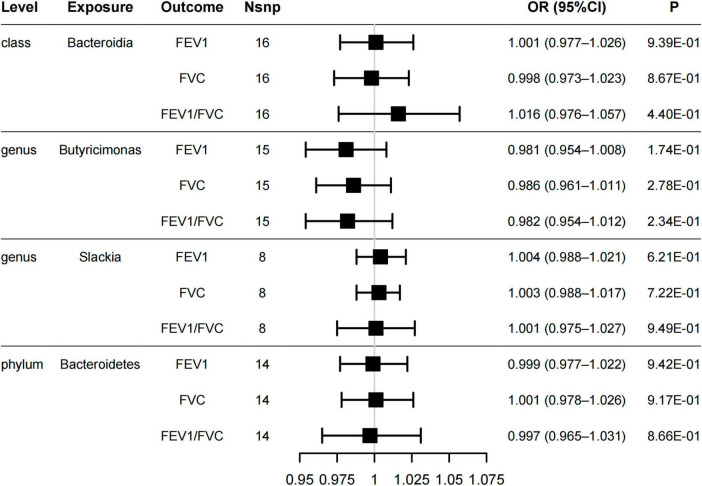
Forest plot of causal relationships estimated for five gut microbiome and lung functions [forced expiratory volume in 1-second (FEV1), forced vital capacity (FVC), and FEV1/FVC ratio] using the inverse variance weighted method.

Lung function is one of the important characteristics for asthma diagnosis, with the FEV1, FVC, and FEV1/FVC ratio being particularly important (Kappen et al., 2023). GINA 2021 (Reddel et al., 2022) highlights the relevance of lung function testing for asthma diagnosis. Therefore, we assessed the associations between the gut microbiome and lung function. The five methods of MR analysis suggested that the genus *Butyricimonas* has no causal effect on FEV1 (OR = 0.981, 95% CI = 0.954–1.008, PIVW = 0.174), FVC (OR = 0.986, 95% CI = 0.961–1.011, PIVW = 0.278) or FEV1/FVC ratio (OR = 0.982, 95% CI = 0.954–1.012, PIVW = 0.234). No evidence was detected indicating a causal relationship between the other four potential gut microbiome and lung function (Figure 3 and Supplementary Table 3). In addition, the sensitivity analysis results supported the robustness of the MR analysis (Supplementary Tables 4, 5).

### 3.3. Causal association of *Butyricimonas* with allergic rhinitis

The prevalence of allergic diseases is increasing worldwide and affects respiratory, digestive, skin, and other systems. However, gut microbiome share a common central role in the risk factors, mechanism, and therapy of these allergic diseases. We evaluated the causal relationship between the five gut microbiome and other allergic diseases, namely, allergic rhinitis, allergic purpura, allergic conjunctivitis, allergic contact dermatitis, and allergic urticaria.

**FIGURE 4:**
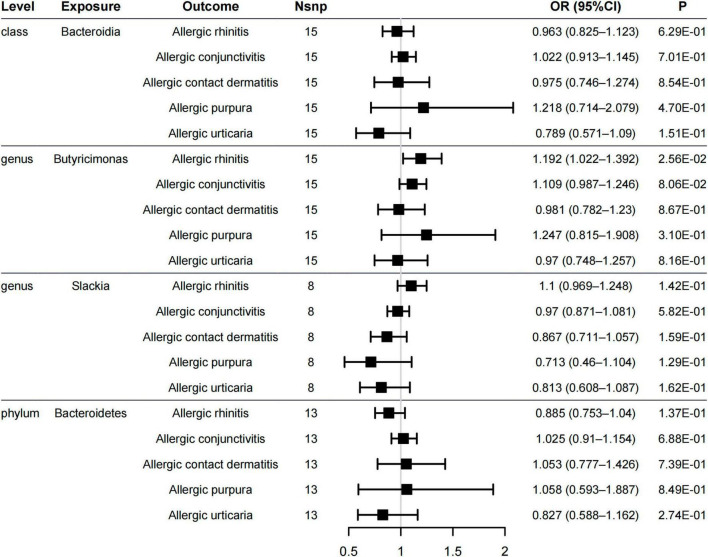
Forest plot of causal relationships estimated for five gut microbiome and other allergic diseases (allergic rhinitis, allergic purpura, allergic conjunctivitis, allergic contact dermatitis, and allergic urticaria) using the inverse variance weighted method.

The genus *Butyricimonas* was associated with allergic rhinitis (OR = 1.192, 95% CI = 1.022–1.392, P_*IVW*_ = 0.0256, Figure 4), but not with allergic purpura (OR = 1.247, 95% CI = 0.815–1.908, P_IVW_ = 0.31), allergic conjunctivitis (OR = 1.109, 95% CI = 0.987–1.246, P_IVW_ = 0.0806), allergic contact dermatitis (OR = 0.981, 95% CI = 0.782–1.23, P_IVW_ = 0.867), and allergic urticaria (OR = 0.97, 95% CI = 0.748–1.257, P_IVW_ = 0.816). Additionally, no significant causality between the other four gut microbiome and other allergic diseases was found in all of the models (Figure 4 and Supplementary Table 6). Neither pleiotropy bias (MR–Egger *p* > 0.1, Supplementary Table 7) or heterogeneity (Cochran’s Q *p* > 0.1, Supplementary Table 8) were found.

## 4. Discussion

This MR study found that the genus *Butyricimonas* was significantly positively associated with a high risk of allergic asthma. In addition, the phylum *Bacteroidetes* and class *Bacteroidia* was also found to have negative associations with the risk of allergic asthma. Genus *Slackia* was identified as having suggestive causal effects with allergic asthma, but more genome-wide data are needed to confirm this result. Therefore, our findings further indicate that gut microbiome has a causal association with allergic asthma, and suggest the regulatory role of gut microbial genera in allergic asthma.

The main metabolite of the genus *Butyricimonas* is butyrate, which is an important energy source for colon cells. Most of the butyrate is metabolized into energy by those cells. Butyrate is believed to be beneficial to the gut, and it helps to promote the body’s anti-inflammatory and immune response, which can ameliorate allergen-induced airway and improve airway hyperresponsiveness in mice (Theiler et al., 2019; Yip et al., 2021). The severity of allergic asthma seems to be related to butyrate produced in the gut. The evidence showed that in the OVA-induced airway inflammation mouse model, supplementing butyrate bacteria from the feces of infants can reduce the airway inflammation (Arrieta et al., 2015). The phylum *Bacteroidetes* and class *Bacteroidia* are colonized bacteria present in the gut of healthy adults. The observational research suggested that the decrease of genus *Butyricimonas* is related to the development of allergic diseases and the increase of the risk of sensitization (Storro et al., 2011; Abrahamsson et al., 2012; Nylund et al., 2013). The phylum *Bacteroidetes* and class *Bacteroidia* has been previously shown to promote the cellular and physical maturation of the developing immune system through its ability to guide the development of CD4^+^T cells, thus inducing the differentiation of Th1 lineage and the correction of Th1/Th2 imbalance (Penders et al., 2007). Interestingly, contrary to the conclusions of observational studies and systematic review (Barcik et al., 2020; Yip et al., 2021) the results of this MR study show that there is a causal association between gut microbiome and allergic asthma or allergic rhinitis. In particular, genus *Butyricimonas* may increase the risk of allergic asthma and allergic rhinitis. The significant change in the proportion of gut microbiome and the increasing risk of allergic asthma seems to occur at the same time.

When establishing the causal effects, multi-levels of research evidence should be considered, but observational studies under different conditions are susceptible to confounding factors, thus reducing the accuracy of conclusions. Therefore, the relevance of observational studies cannot be equated with causal correlations. MR avoids the influence of confounding factors through genetic instrumental variables, and it can carry out accurate causal assessment. However, the development of allergic diseases is a long-term process, and more data are needed to support our findings.

Our study is the first MR analysis of gut microbiome, allergic asthma, and multiple allergic diseases. It has several advantages: First, compared with the inherent limitations of observational studies, MR studies are unlikely to be affected by reverse causality and confounding factors. Second, extensive GWAS sample data, two sets of independent IVs, and different methods are applied to causal association assessments to improve reliability. It must be acknowledged that this study also has the following limitations: First, this study is limited to individuals of European descent, and the difference in dietary habits lead to diversity of gut microbiome among different populations, so it may be limited to extrapolate the research findings to other ethnic groups. Second, this study is unable to determine whether overlapping participants were included in the exposure and outcome GWAS used in the two-sample MR analysis. Lastly, this study cannot further answer the different role the genus *Butyricimonas* played in this MR study and other observational studies.

In conclusion, this MR study shows there are causal associations between gut microbiome and allergic asthma in European populations, as well as a potential causal association between gut microbiome and allergic rhinitis. Several types of gut microbiome were found to be pathogenic or protective factors of allergic asthma, and may have the prospects for the prevention and treatment of allergic asthma. With this research we hope to strengthen the understanding of the characteristics of gut microflora.

## Data availability statement

The original contributions presented in this study are included in the article/Supplementary material, further inquiries can be directed to the corresponding author.

## Author contributions

WW, YQ, and CY designed the research. WW, YQ, XH, and YR collected and analyzed the data. YQ, XH, and YR performed the literature search. WW and CY drafted the manuscript. YQ and AP supervised the study. All authors were involved in writing the manuscript, contributed to the article and approved the submitted version.
